# From the Gut to the Heart: Campylobacter jejuni Enteritis Leading to Myopericarditis

**DOI:** 10.7759/cureus.1326

**Published:** 2017-06-09

**Authors:** Faisal Inayat, Nouman Safdar Ali, Iqra Riaz, Hafeez Ul Hasan Virk

**Affiliations:** 1 Department of Medicine, New York-Presbyterian Hospital, Weill Cornell Medical College, New York City, NY, USA; 2 Department of Medicine, Jinnah Hospital, Allama Iqbal Medical College, Lahore, Pakistan; 3 Department of Medicine, Mayo Hospital, King Edward Medical University, Lahore, Pakistan; 4 Department of Medicine, St. Luke’s-Roosevelt Hospital Center, Icahn School of Medicine at Mount Sinai, New York City, New York

**Keywords:** campylobacter jejuni, enteritis, myocarditis, cardiomyopathy, myopericarditis

## Abstract

*Campylobacter jejuni* enteritis is the most common enteric infection in the developed world. Although rare, pericarditis and myopericarditis have been increasingly documented as complications following campylobacteriosis. The present paper implicates that myopericarditis and non-ischemic cardiomyopathy following *Campylobacter jejuni* infection might be more prevalent than initially thought and perhaps underreported so far. Therefore, it is imperative to perform the appropriate initial diagnostic testing, including stool cultures, in order to make an accurate diagnosis early in the course of the disease. Identifying the etiology of myopericarditis as bacterial will ensure appropriate treatment with antibiotics in addition to the cardiac medications needed for supportive care.

## Introduction

*Campylobacter jejuni* is a common cause of enterocolitis. Recently, myocarditis and non-ischemic cardiomyopathy were highlighted as possible complications of *C. jejuni* infection [1]. These patients can pose a diagnostic and therapeutic challenge. However, if the physician is aware of this rare complication, the clinical diagnosis of bacterial myocarditis can be confirmed by specific radiological findings in combination with the positive stool cultures for *C. jejuni *and the need for endomyocardial biopsies can be minimized.

The present study discusses a recently treated patient presenting with non-ischemic cardiomyopathy who ultimately was diagnosed with *C. jejuni* infection. Furthermore, it implicates that prompt infection control can oppose the pathogenetic mechanisms that damage the myocardium and may prevent arrhythmia, dilated cardiomyopathy, and irreversible loss of ventricular function. The role of adjunctive immunosuppressive therapy remains unclear.

Hence, the diagnosis of myocarditis or pericarditis should be considered whenever symptoms of relatively acute chest pain, palpitations or shortness of breath are encountered with raised cardiac enzymes and with little evidence of coronary artery disease, especially in a young individual like this patient. In the published literature, the number of cases of myocarditis or myopericarditis related to *C. jejuni* is scarce.

## Case presentation

A 20-year-old male presented to the Mount Sinai St. Luke's emergency department (ED) after he woke up with severe chest pain in the morning. The patient was on a trip to Panama for a week where he had an episode of fever (102 °F) with nausea, vomiting, and watery diarrhea for three days. He developed fatigue, generalized myalgia, a cold sore on the lower lip, and a vesicular rash on the left forehead. He also reported some mosquito bites. One of his family members had been having similar symptoms about two weeks before his ailment.

The patient flew back to New York where his primary care physician prescribed oral ciprofloxacin 500 mg twice daily and his stool studies were ordered. The diarrhea stopped, so he went to the gym and on his way back on the bicycle, he felt some irregular heartbeats which went away in 10 minutes. The next morning, he woke up with sudden, severe, left-sided chest tightness and pain. The pain was non-radiating and pleuritic in nature that got worse with deep breaths and improved with leaning forward. He denied shortness of breath, diaphoresis, abdominal pain, and dizziness. The patient had no family history of premature cardiac disease.

**Figure 1:**
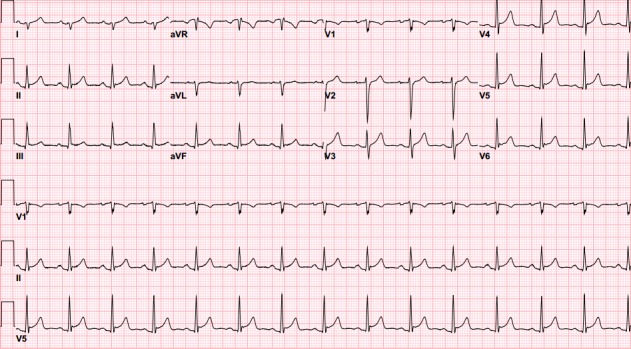
Electrocardiogram The initial electrocardiogram showing right axis deviation with J-point early depolarization mimicking ST-segment elevation.

In the ED, his electrocardiogram showed right axis deviation with J-point early depolarization mimicking ST elevation (Figure 1).

The laboratory studies were within normal limits, except troponins and creatine kinase (CK)-MB that were elevated to 5.7 ng/mL (normal <0.03 ng/mL) and 54.7 ng/mL (normal <7.3 ng/mL), respectively. He was managed conservatively with analgesics, antacids, and fluids. The patient was admitted to the cardiac care unit for further workup.

**Figure 2:**
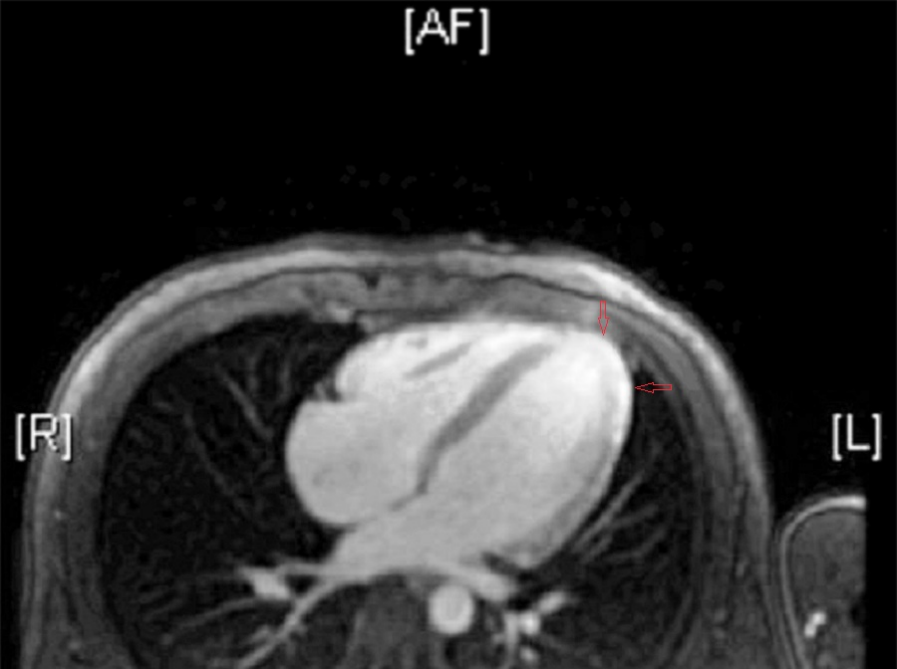
Cardiac MRI Direct axial image of cardiac MRI showing sub-epicardial enhancement in basal to apical anterolateral, inferiolateral and anteroseptal wall. MRI - magnetic resonance imaging.

Transthoracic echocardiography (TTE) was performed. It showed an ejection fraction (EF) of 55% with normal ventricular wall motions. Since his risk for coronary artery disease was low, computed tomography (CT) of the coronary arteries was done and that showed normal coronaries and a calcium score of 0. On cardiac magnetic resonance imaging (MRI), sub-epicardial enhancement in apical anterior, inferior and true apex wall was noted. Sub-epicardial enhancement in basal to apical anterolateral, inferolateral and anteroseptal wall was present (Figure 2).

The left ventricle was mildly dilated with mid-systolic dysfunction (LVEF, 41%), and extensive myocardial late gadolinium enhancement was noted in a non-ischemic pattern, which was most consistent with myopericarditis.

The treatment was initiated with aspirin 325 mg orally once a day and colchicine 0.6 mg twice daily. On day 2 of admission, troponins trended up to 39 ng/mL (normal <0.03 ng/mL), CK-MB to 99.4 ng/mL (normal <7.3 ng/mL), and CK to 2020 U/L (normal 30-170 U/L).

Subsequently, the patient reported resolution of the chest pain. The erythrocyte sedimentation rate (ESR) was 18 mm/hr (0-13 mm/hr) and C-reactive protein (CRP) 121 mg/L (normal <5.1 mg/L). Repeat TTE showed no accumulation of fluid around the pericardium. The blood parasite screen was negative. Serologies for Coxsackie virus, *Toxoplasma gondii*, Cytomegalovirus (CMV), Parvovirus B19, human immunodeficiency virus (HIV), and hepatitis C came back inconclusive. Antinuclear antibody (ANA), anti-ds DNA, and rheumatoid factor were negative. Continuous telemetry monitoring showed some runs of non-sustained ventricular tachycardia (NSVT). Oral metoprolol 25 mg twice daily was started for that. He remained clinically stable over the rest of the hospital course.

The patient’s primary care physician was contacted for the results of his stool cultures collected at the time of his pre-hospitalization visit. The growth of *C. jejuni* was confirmed. Infectious disease service was consulted for *C. jejuni* treatment and they recommended continuing aspirin and colchicine therapy with weekly ESR and CRP monitoring until they are normalized. The patient was discharged on metoprolol succinate 50 mg/day and follow-up appointments with cardiology and infectious disease service. On close follow-up, his ESR and CRP normalized after six weeks of treatment, so aspirin and colchicine therapy was stopped.

## Discussion

Campylobacter species lead to most of the small bowel infections in the developed world. Early complications of *C. jejuni* infection include cholecystitis, peritonitis, urticaria, erythema nodosum, and abdominal aortic septic pseudoaneurysm. Reactive arthritis and Guillain-Barré syndrome can also develop later in the course of the disease. Campylobacter infection can also lead to bacteremia but it is exceedingly rare in immunocompetent individuals. Albeit rare, it may cause pericarditis and/or myocarditis [1]. The incidence of myopericarditis in confirmed cases of *C. jejuni*-associated enterocolitis is reportedly low. It was 0.4% in a study conducted at the University of Helsinki based on subjective data of patients’ symptom recall [2].

The mechanism by which *C. jejuni*causes myopericarditis remains to be determined. In literature, the two hypotheses put forward so far suggest immune-mediated injury to the cardiac tissue or direct infection of myopericardium with the bacterium [3]. Most of the immune-mediated responses take time to build up in the course of infection. Histopathological analysis of the myocardial tissue in the first reported death as a result of myocarditis with* C. jejuni*enteritis revealed marked neutrophilic infiltration and inflammatory changes that were different from those seen in cases of viral myocarditis. Florid acute myocarditis with no detection of *C. jejuni* on polymerase chain reaction (PCR) studies indicated the lesser role of direct invasion and the greater role of the toxins from the infective organism. The present patient initially reported irregular heartbeat and chest discomfort within 24 hours of the infection that is suggestive of direct bacterial invasion.

Diagnosis is based on the clinical presentation and the laboratory confirmation of the *C. jejuni*, either on stool culture or biopsy. Serological tests are usually of little use in acute infection as antibodies take time to build up and become detectable in serum. Cardiac MRI showing subepicardial and/or myocardial enhancement can provide significant confirmation of the myopericardial inflammation, but its sensitivity remains low [2-4].

Although endomyocardial biopsy is the gold standard for diagnosing myocarditis, it was not performed in this patient because of strong temporal relationship with the infection and lack of evidence of dilated cardiomyopathy or vascular compromise. A biopsy is indicated when severe heart failure is encountered and benefits of the biopsy outweigh the risks [5]. However, histopathological analysis of tissue specimen can be inconclusive because of variability in the distribution of inflammatory changes throughout the myopericardium and it can be considered a reason to avoid biopsy in uncomplicated cases.

To date, most of the reported cases of myocarditis or myopericarditis were of young males who recovered fully with conservative treatment [1-5]. The present patient had typical acute gastroenteritis, diaphoresis, chest tightness, raised cardiac enzymes, and positive stool cultures with little chance of any alternative explanation, so the diagnosis of *C. jejuni-*related myopericarditis was probed. Our patient had some palpitations on the day before coming to the medical emergency that may suggest transient arrhythmia. Previously, atrial fibrillation has been reported in multiple cases with Campylobacter infection [5].

Acute management involves stabilization of the cardiac tissue perfusion and avoidance of electrolyte imbalance. Electrolyte imbalance can predispose to other cardiovascular problems, including ventricular fibrillation that can be fatal if not reversed in time. Extracorporeal membrane oxygenation (ECMO) can be utilized in cases of severe cardiogenic shock [6]. Angiotensin-converting enzyme (ACE) inhibitors are used to decrease heart failure-related morbidity and mortality in such patients. Macrolides and fluoroquinolones cover most of the Campylobater infections in an effective way, but in immunocompromised and elderly cases, it is necessary to be vigilant with resistant bacteria. Colchicine is used in most cases of pericarditis to decrease the degree of inflammation. Prognosis of myopericarditis has been reported to be worse with pre-existing conditions such as cardiomyopathies and congenital syphilis [7-8].

FoodNet Population Surveys conducted in 2010 at 10 different US state health sites reported a total of 19,089 laboratory-confirmed cases of active foodborne infections, and 6,365 were caused by Campylobacter species. Campylobacter infections were the most common with an incidence of 13.6 per 100,000. Shigella and Cryptosporidium infections followed [9]. These are preventable conditions and the risk of these infections increases in immunocompromised states. The number of *C. jejuni* infections is increasing worldwide; cardiac complications, although rare, are a remarkable manifestation of this pathogen and should be always kept in mind.

## Conclusions

*C. jejuni*-associated cardiomyopathy has increasingly been recognized in clinical practice. Its clinical evolution and manifestations are mostly benign, yet may culminate in severe heart failure, arrhythmias, and death. Therefore, timely utilization of resources for detection of such life-threatening complications related to Campylobacter is essential. A high index of clinical suspicion is imperative when patients present with acute heart disease following or accompanying an episode of severe enterocolitis.
